# Red blood cell, hemoglobin and heme in the progression of atherosclerosis

**DOI:** 10.3389/fphys.2014.00379

**Published:** 2014-10-02

**Authors:** Viktória Jeney, György Balla, József Balla

**Affiliations:** ^1^Department of Medicine, University of DebrecenDebrecen, Hungary; ^2^MTA-DE Vascular Biology, Thrombosis and Hemostasis Research Group, Hungarian Academy of SciencesDebrecen, Hungary; ^3^Department of Pediatrics, University of DebrecenDebrecen, Hungary

**Keywords:** atherosclerosis, intraplaque hemorrhage, red blood cell lysis, hemoglobin oxidation, haptoglobin, hemopexin, heme-oxygenase, ferritin

## Abstract

For decades plaque neovascularization was considered as an innocent feature of advanced atherosclerotic lesions, but nowadays growing evidence suggest that this process triggers plaque progression and vulnerability. Neovascularization is induced mostly by hypoxia, but the involvement of oxidative stress is also established. Because of inappropriate angiogenesis, neovessels are leaky and prone to rupture, leading to the extravasation of red blood cells (RBCs) within the plaque. RBCs, in the highly oxidative environment of the atherosclerotic lesions, tend to lyse quickly. Both RBC membrane and the released hemoglobin (Hb) possess atherogenic activities. Cholesterol content of RBC membrane contributes to lipid deposition and lipid core expansion upon intraplaque hemorrhage. Cell-free Hb is prone to oxidation, and the oxidation products possess pro-oxidant and pro-inflammatory activities. Defense and adaptation mechanisms evolved to cope with the deleterious effects of cell free Hb and heme. These rely on plasma proteins haptoglobin (Hp) and hemopexin (Hx) with the ability to scavenge and eliminate free Hb and heme form the circulation. The protective strategy is completed with the cellular heme oxygenase-1/ferritin system that becomes activated when Hp and Hx fail to control free Hb and heme-mediated stress. These protective molecules have pharmacological potential in diverse pathologies including atherosclerosis.

## Introduction

Complications of cardiovascular disease, and in particularly luminal thrombosis triggered by rupture of atherosclerotic lesions, are the leading cause of mortality and morbidity worldwide. Not all the plaques are prone to rupture, only the vulnerable ones, characterized by thin fibrous cap. Recently, plaque neovascularization and intraplaque hemorrhage (IPH) have been linked to plaque progression and vulnerability and these processes gained substantial interest (reviewed in Moreno et al., 2012).

In this review we briefly summarize what is known regarding the triggers of neovascularization and IPH. We overview the fate of red blood cells (RBCs) in the highly oxidative environment of the atherosclerotic plaque, and discuss the defense and adaptation mechanisms which have evolved to control the deleterious effects of cell free Hb.

### Neovascularization in atherosclerotic lesions

Oxygen and nutrients are diffused from the vessel lumen into the intimal and medial cells of healthy vessels, while the outer layers of media and the adventitia are nurtured by the capillary network of vasa vasorum (Moreno et al., 2006, 2012). Neovascularization, that is the growth of capillary-like microvessels into the thickened media and intima, has long been considered as a prominent feature of late-stage atherosclerotic plaques (O'Brien et al., 1994). Nowadays growing evidence support that in fact neovascularization is present in early atherosclerotic lesions (Jeziorska and Woolley, 1999) particularly when the thickness of the tunica intima exceeds the maximum oxygen diffusion distance that is ~200–250 μm (Geiringer, 1951; Torres Filho et al., 1994; Moulton et al., 1999).

#### Hypoxia as a trigger of plaque neovascularization

Hypoxia, a condition when oxygen tension drop below its normal level in the particular tissue (20–100 mmHg), is a long-recognized stimulus for angiogenesis (Knighton et al., 1983). Using oxygen microelectrodes or specific hypoxia markers, hypoxia of the mid-region of the atherosclerotic plaques was demonstrated in various animal models (Jurrus and Weiss, 1977; Zemplenyi et al., 1989; Crawford and Blankenhorn, 1991; Bjornheden et al., 1999). In humans, the presence of hypoxic milieu in advanced atherosclerotic lesions of carotid arteries was also shown (Sluimer et al., 2008). As a consequence of hypoxia, switch from aerobic to anaerobic metabolism, characterized by glucose and ATP depletion and lactate accumulation, occurs in both human and experimental atheroma (Levin et al., 2003; Leppanen et al., 2006). Recently it has been shown that both sustained and intermittent hypoxia accelerates the progression of atherosclerosis in apolipoprotein E (apoE) deficient mice (Nakano et al., 2005; Jun et al., 2010).

Hypoxia-inducible factor-1 (HIF-1) pathway is the major mediator of the biological effects of hypoxia (Wang and Semenza, 1995). HIF-1 is active exclusively as a heterodimer of HIF-1α and HIF-1β subunits. While HIF-1β is stable, the level of HIF-1α is regulated by oxygen (Wang et al., 1995). Under normoxia, HIF-1α subunits are hydroxylated by the Fe^2+^-dependent prolyl hydroxylases (PHD) followed by ubiquitination and subsequent degradation by the proteasome (Maxwell et al., 1999; Ivan et al., 2001). In contrast, under hypoxia PHDs are inactive and HIF-1α subunits are no longer degraded. This allows the formation of the active HIF-1 heterodimer, which then translocate into the nucleus, binds to the hypoxic response elements and initiates transcription of target genes (Wenger et al., 2005). These genes are involved in the adaptation of the organism to hypoxic condition, such as vascular endothelial growth factor (VEGF) that has a pivotal role in angiogenesis (Forsythe et al., 1996).

Expression of HIF-1α is increased in deep and less-vascularized layers of human carotid and femoral endarterectomy specimens (Vink et al., 2007; Higashida et al., 2008). Increased HIF-1 alpha expression is associated with elevated level of VEGF suggesting that HIF-1 pathway is active and most probably play a role in neoangiogenesis in these hypoxic regions of the atherosclerotic plaques (Vink et al., 2007; Higashida et al., 2008; Gao et al., 2012).

#### Inflammation and ROS as triggers of plaque neovascularization

Although hypoxia is by far the most studied angiogenic factor, recent discoveries highlighted the role of reactive oxygen species (ROS) that are implicated in both physiological and pathological angiogenesis under normoxic conditions (reviewed in Kim and Byzova, 2014). ROS activates the HIF-1/VEGF pathway that serves as the major underlying mechanism of ROS-mediated angiogenesis. Additionally, recent discoveries highlighted the role of toll-like receptors (TLR) behind angiogenic activity of ROS. The activation of various TLR receptors (TLR2, TLR3, TLR4, TLR2/6) can lead to angiogenesis in both HIF-1/VEGF-dependent and HIF-1/VEGF-independent manners (Leibovich et al., 2002; Pollet et al., 2003; Grote et al., 2010; Paone et al., 2010; Spirig et al., 2010) (reviewed in Bordon, 2010). For example activation of TLR4 by lipopolysaccharide activates the HIF-1 pathway (Vink et al., 2007), whereas activation of TLR2 by its novel endogenous ligand, ω-(2-carboxyethyl) pyrrole, leads to an angiogenic response that is independent of VEGF (West et al., 2010).

Besides its direct angiogenic potential, ROS have been implicated in the generation of lipid oxidation products with proangiogenic activities, such as oxidized phospholipids that can be found in large amounts in atherosclerotic lesions (Bochkov et al., 2006; West et al., 2010; Hutter et al., 2013).

#### Physiological and pathological angiogenesis

Angiogenesis in general is fundamental for development and repair. It was proposed that physiologic angiogenesis can serve as a defense mechanism in atherosclerosis to compensate tissue hypoxia and restore homeostasis in the vessel wall (Moreno et al., 2006). Theoretically neovessels could provide channels for immune cells and bone marrow-derived progenitors to resolve inflammation and facilitate repair of the diseased vessel, respectively. It was also postulated that physiological angiogenesis contributes to the elimination of accumulated lipids from the intima (Moreno et al., 2006). Regardless of these potential beneficial effects, growing body of evidence suggest that plaque neovascularization correlates with the progression of atherosclerosis and neovessel density was found to be an independent risk factor for aortic plaque rupture (McCarthy et al., 1999; Moreno et al., 2004). Many studies revealed that inhibition of angiogenesis with different approaches reduces plaque growth (Moulton et al., 1999, 2003; Luttun et al., 2002; Petrovan et al., 2007; Drinane et al., 2009; Bot et al., 2011), whereas stimulation of angiogenesis with VEGF or nicotine results in elevated lesion progression in experimental atherosclerosis (Celletti et al., 2001; Heeschen et al., 2001). The observed disadvantageous effects of plaque neovascularization might be explained by pathological angiogenesis that proceeds in an uncontrolled manner, and results the formation of an abnormal neovessel structure.

Neovessles can originate from three sources. Sprouting of the adventitial vasa vasorum in response to angoigenic stimuli is the most widely accepted mechanism of neovessel formation. Besides vasa vasorum, luminal endothelial cells, or recruitment and differentiation of vascular progenitor cells inside the plaque can be involved in the formation of neovessels (reviewed in Galis and Lessner, 2009). Regardless of their origin, plaque neovessels differ both anatomically and in their response to different stimuli from the normal vessels (Ritman and Lerman, 2007). Neovessels are dysmorphic and characterized by discontinuous basement membrane and a relatively low number of tight junctions between endothelial cells (Heistad et al., 1981; Dunmore et al., 2007; Sluimer et al., 2009). Moreover these premature vessels are relatively poor in smooth muscle cells or pericytes (Kolodgie et al., 2003). Consequently, neovessels are leaky and unable to control intraluminal pressure therefore they are prone to rupture (Zhang et al., 1993; Sluimer et al., 2009).

### Intraplaque hemorrhage

Continuous leakage or rupture of immature neovessels leads to extravasation of RBCs within plaques which process is defined as IPH. IPH is present in about 40% of high-risk plaques (Kockx et al., 2003). Recently IPH has been linked to plaque progression and vulnerability and nowadays is considered as a critical event in triggering atherosclerosis-associated acute clinical symptoms (Michel et al., 2011). Different theories evolved about the molecular mechanisms via which IPH contribute to plaque progression.

#### RBC membrane-derived cholesterol as a trigger for lipid core expansion and inflammation

The casual relationship between elevated cholesterol level and atherosclerosis is known for more than 60 years. Early atherosclerotic lesions are characterized by subendothelial accumulation of cholesterol-laden macrophages called foam-cells. During plaque progression foam cells dye and release free cholesterol that deposits inside the plaque forming the necrotic core a characteristic feature of more advanced lesions (Lusis, 2000). For decades low-density lipoprotein (LDL) was considered as the main source of atherosclerotic plaque lipid content, and lowering circulating LDL-cholesterol level is still a major approach for anti-atherosclerotic therapies (Sahebkar and Watts, 2013).

Recently it has been shown that in human atherosclerotic lesions cholesterol crystals are co-localized with glyophorin A, a characteristic protein of RBC membrane, suggesting that cholesterol content of RBC membrane contributes to lipid deposition and lipid core expansion upon IPH (Kolodgie et al., 2003, 2007). In fact, RBC membrane is particularly abundant in cholesterol (Yeagle, 1985). RBCs are not able to synthetize lipids, but there is an active exchange between RBC membrane lipids and plasma lipoproteins. Therefore lipid composition of RBC membrane reflects plasma lipoprotein levels. For example it has been shown that familial hypercholesterolemia is associated with elevated RBC membrane-associated cholesterol (Koter et al., 2002) and that high-fat diet increase membrane lipid content of RBCs in experimental animal models (Bhandaru et al., 1982; Ivanov et al., 1991; Tziakas et al., 2013). Accordingly, lipid lowering strategies such as statin treatment and life-style changes have been shown to positively modulate RBC lipid composition which might contribute to the atheroprotective effects of these approaches (Tynan et al., 1995; Koter et al., 2002; Caspar-Bauguil et al., 2010; Tziakas et al., 2013).

The direct evidence that RBC contribute to lesion progression is provided by the experiment of Kolodgie et al. in which they injected packed RBCs directly into quiescent atherosclerotic lesions in rabbit aortas. RBC injection triggered the enlargement of necrotic core and formation of free cholesterol crystals along with excessive macrophage infiltration (Kolodgie et al., 2003).

Inflammation has a fundamental role in mediating all stages of atherosclerosis (Libby, 2002). Discoveries of the last 20 years made us to understand that besides pathogen-associated molecular patterns (PAMPs) several endogenous molecules, called danger- or damage-associated molecular patterns (DAMPs) can activate cellular receptors leading to downstream inflammation (Matzinger, 1994, 2002). Rajamaki et al. showed that cholesterol crystals serve as DAMPs and cause the activation of the NLRP3 [nucleotide-binding domain leucine-rich repeat containing (NLR) family, pyrin domain containing 3] inflammasome in macrophages (Rajamaki et al., 2010). Activation of NLRP3 inflammasome by cholesterol crystals leads to the activation of cytoplasmic caspase-1 that promotes maturation and secretion of the proinflammatory cytokine IL-1β (Rajamaki et al., 2010) and thus link altered cholesterol metabolism and inflammation in atherosclerotic lesions.

#### RBC lysis, Hb release and Hb oxidation upon IPH

**Figure 1:**
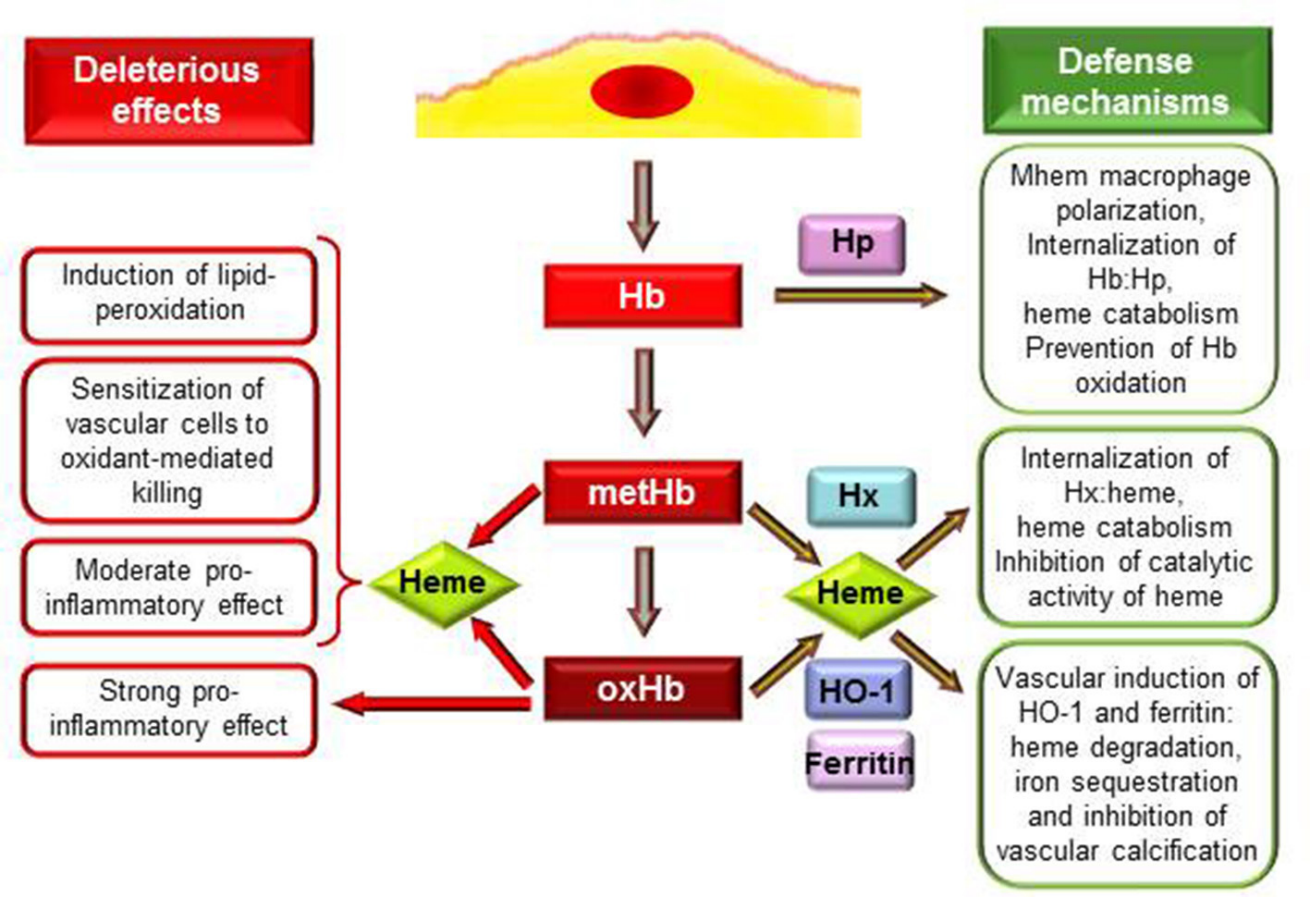
**Deleterious effects and defense mechanisms triggered by extracellular hemoglobin and its oxidation products upon intraplaque hemorrhage**. Interactions between RBCs and plaque lipids lead to lysis of erythrocytes and release of Hb. Extracellular Hb by reacting with plaque lipids undergo rapid oxidation to metHb and oxHb. MetHb and oxHb release their heme moieties that possess pro-oxidant and pro-inflammatory properties. OxHb is a strong pro-inflammatory agonist which effect is independent of heme release. As an atheroprotective mechanism in response to Hb stress Mhem macrophages polarization occurs. Hp binding Hb but not oxHb attenuates Hb oxidation and their uptake by Mhem macrophages results in heme catabolism. Heme liberated from MetHb and oxHb can be captured by hemopexin (Hx). Hx inhibits catalytic activity of heme, and after internalization of Hx:heme complex heme is catabolized. Induction of heme oxygenase-1 (HO-1) and ferritin in vascular cells in response to such and insult (Hb, oxHb, heme, lipid oxidation) provides inhibition of oxidant damage and inflammation. HO-1 degrades heme into biliverdin, CO and iron which is sequestered by ferritin. Products of HO-1 mediated heme degradation: CO and biliverdin—that is readily converted to bilirubin—possess different antioxidant and anti-inflammatory properties. Additionally, ferritin inhibits vascular calcification.

While compartmentalized in RBCs oxidation of Hb is controlled by a highly effective antioxidant defense system including enzymatic (Cu/Zn superoxide dismutase, catalase, glutathione peroxidase, and peroxiredoxins) and non-enzymatic (glutathione) scavengers (Siems et al., 2000; Jeney et al., 2013). Upon IPH RBCs enter to the highly oxidative milieu of atherosclerotic lesion, the “death zone” that contains cytotoxic products of lipid peroxidation such as lipid hydroperoxides, aldehydes, and carbonyls (Li et al., 2006). The high occurrence of IPH prompted us to study the interaction of RBC and atheroma lipids. We revealed that these reactive lipids, extracted from human atheroma trigger the lysis of RBCs (Figure 1) (Nagy et al., 2010). Oxidized LDL and cumene hydroperoxide mimic the effect of plaque lipid extract on RBC lysis (Nagy et al., 2010). Moreover, enzymatic conversion of lipid-hydroperoxides to lipid-alcohols by glutathione/glutathione peroxidase causes significant inhibition of RBC lysis triggered by oxLDL and plaque lipids highlighting the critical role of lipid-hydroperoxides in RBC lysis (Nagy et al., 2010).

Hb once outside the protective environment of RBC is prone to oxidation (Figure 1). Auto-oxidation of Hb occurs resulting in metHb generation meanwhile superoxide anions are formed (Table 1, equation 1). Peroxides, such as H_2_O_2_ can trigger a two-electron oxidation of Hb leading to the formation of ferryl (Fe^4+^ = O^−^_2_) Hb (Table 1, equation 2), whereas the reaction of metHb with H_2_O_2_ yields ferrylHb radical (Hb^·+^(Fe^4+^ = O^−^_2_)) in which the unpaired electron is associated with the globin or the porphyrin ring (Table 1, equation 3) (Harel and Kanner, 1988; Patel et al., 1996; Alayash et al., 2001; Jia et al., 2007).

**Table 1:** **Routes of hemoglobin oxidation**.

	**Formed species**
Hb(Fe^2+^)O_2_ → Hb(Fe^3+^) + O^•−^_2_	Methemoglobin
Hb(Fe^2+^)O_2_ + H_2_O_2_ → Hb(Fe^4+^ = O^−^_2_) + H_2_O + O_2_	Ferrylhemoglobin
Hb(Fe^3+^) + H_2_O_2_ → Hb^•+^(Fe^4+^ = O^−^_2_) + H_2_O	Ferrylhemoglobin globin radical
Hb(Fe^4+^ = O^−^_2_) + 2H^+^ → Hb^•+^(Fe^3+^) + H_2_O	Methemoglobin globin radical
Hb^•+^(Fe^3+^) + Hb^•+^(Fe^3+^) → (Fe^3+^) ^+^Hb-Hb^+^(Fe^3+^)	Covalently cross-linked methemoglobin multimer

Auto-oxidation of Hb generates metHb and superoxide anions (equation 1). H_2_O_2_ triggers a two-electron oxidation of Hb leading to the formation of ferryl (Fe^4+^ = O^−^_2_) Hb (equation 2). The reaction of metHb with H_2_O_2_ yields ferrylHb radical (Hb^·+^(Fe^4+^ = O^−^_2_)) in which the unpaired electron is associated with the globin or the porphyrin ring (equation 3). FerrylHb can trigger further production of globin radicals via an intramolecular electron transfer between the ferryl iron and specific amino acid residues of the globin chains resulting the formation of metHb globin radical (equation 4). Termination reactions of globin- and porphyrin-centered radicals lead to the formation of globin-globin (equation 5) crosslinks.

The generated high-valence iron compounds are highly reactive intermediates that can decay by several routes (Reeder et al., 2008). FerrylHb can trigger further production of globin radicals via an intramolecular electron transfer between the ferryl iron and specific amino acid residues such as αTyr-24, αTyr-42, αHis-20, βTyr-35, βTyr-130, and βCys-93 of the globin chains resulting the formation of metHb globin radical (Table 1, equation 4) (Ramirez et al., 2003; Deterding et al., 2004; Jeney et al., 2013). Termination reactions of globin- and porphyrin-centered radicals lead to the formation of globin-globin (Table 1, equation 5) or porphyrin-globin crosslinks. The common feature of these structurally heterogeneous molecules is the modification of the globin chain. The nomenclature of these molecules is not concise in these days. Nevertheless, along this review in order to distinguish from metHb and ferrylHb—in which only the oxidation state of heme iron is altered but no globin modification is present—we will refer to those globin-modified molecules as oxidatively modified Hb (oxHb).

Studying the interaction of Hb and atheroma lipids, we observed a severe oxidation of Hb leading to the generation of metHb and oxHb (Figure 1). Moreover, we revealed significant accumulation of metHb and oxHb within human complicated atherosclerotic lesions—covalently cross-linked globin-globin multimers, and dityrosine formation occurs upon IPH—suggesting that the above-mentioned reactions take place in such lesions (Nagy et al., 2010; Jeney et al., 2013). We suggested that oxidation of Hb in the atherosclerotic plaque might be triggered by reactive lipid mediators (Figure 1). Besides atheroma lipids oxLDL was also shown to cause oxidation of cell-free Hb, producing metHb as well as ferrylHb and oxHb (Tynan et al., 1995; Nagy et al., 2005; Potor et al., 2013). Oxidation of Hb provoked by reactive lipid mediators can be inhibited by the heme scavenging Hx and by the elimination of lipid hydroperoxides, suggesting that interactions between the heme moiety and the hydroperoxide group drive the oxidation (Jeney et al., 2013).

#### Extracellular Hb, oxidized Hb species and heme as triggers of lipid peroxidation and endothelial damage

Oxidative modification of LDL and endothelial damage are key elements of atherogenesis. More than 20 years ago Balla et al. showed that heme, the prosthetic group of Hb, is a very efficient trigger of LDL oxidation *in vitro* and suggested that it might be a physiological mediator of LDL oxidation *in vivo* (Balla et al., 1991a). We also showed that heme greatly amplifies oxidant-mediated endothelial damage (Balla et al., 1990, 1991b). Several lines of evidence support, that these heme-triggered events have etiopathogenic roles in diverse vascular pathologies, including atherosclerosis (Balla et al., 2007). Deficiency of the heme-catabolizing enzyme, heme oxygenase-1 (HO-1), in humans was found to be associated with elevated plasma heme levels, extensive LDL oxidation, severe endothelial damage and accelerated atherosclerosis (Yachie et al., 1999; Jeney et al., 2002; Kawashima et al., 2002; Radhakrishnan et al., 2011). The role of HO-1 in atherogenesis was also examined in animal models. It has been shown that overexpression of HO-1 in apoE deficient mice inhibit lesion formation (Juan et al., 2001), whereas HO-1 deficiency is associated with accelerated atherosclerosis in apoE deficient mice (Yet et al., 2003). In heme-mediated LDL oxidation a unique oxidation product, 5-hydroxy-2-amino valeric acid (HAVA) is formed (Julius and Pietzsch, 2005). HAVA is a hallmark of heme-mediated LDL oxidation, because other known triggers of LDL oxidation, such as HOCl, H_2_O_2_ alone or in combination with Cu^2+^ or Fe^2+^ induce no or minor HAVA formation (Julius and Pietzsch, 2005). HAVA levels in LDL was found to be elevated in patients with impaired glucose tolerance and with diabetes mellitus suggesting that heme-mediated LDL oxidation occurs in these patients (Julius and Pietzsch, 2005).

Not only free heme, but metHb and oxHb trigger LDL oxidation and sensitize endothelial cells to oxidant-mediated killing (Balla et al., 1993; Paone et al., 2010; Potor et al., 2013). These Hb species readily release heme moiety (Bunn and Jandl, 1968), which step is of crucial importance in mediating their effect (Figure 1). This notion is supported by the observation that restriction of heme release using different approaches, such as binding of Hb to Hp or strengthening the globin-heme binding, inhibits the deleterious effects of these Hb species (Balla et al., 1993; Jeney et al., 2002; Nagy et al., 2010; Potor et al., 2013).

#### Extracellular Hb, oxidized Hb species and heme as modulators of inflammation

Inflammation is an important etiopathogenic component of atherogenesis, and several evidence suggest that cell free Hb, oxidized Hb species and heme possess specific immunomodulatory activities (Figure 1). Hemolytic or hemorrhagic episodes are often associated with inflammation even in the absence of infectious agents (Arruda et al., 2005; Gram et al., 2013). Vascular endothelium, that provides a barrier between blood and tissue has a critical role in the inflammatory response mainly by inducing the leukocyte adhesion cascade to facilitate transmigration of inflammatory cells to the inflamed tissue. Endothelial cells when exposed to heme or oxHb up-regulate the expression of adhesion molecules: intracellular adhesion molecule-1 (Icam-1), vascular cell adhesion molecule-1 (Vcam-1) and E selectin (Wagener et al., 1997; Silva et al., 2009). Comparing to heme, oxHb is a more robust inducer of this inflammatory response, as one-tenth of oxHb provoke the same response as heme. Also, the mechanism of oxHb-triggered inflammatory response seems to be different from the one that heme initiates. OxHb mediated inflammatory response is independent of heme release, which notion is supported by the observation that metHb that can release heme moiety similarly to oxHb has no pro-inflammatory properties (Figure 1) (Silva et al., 2009). Additionally, endothelial cells exposed to oxHb show rearrangement of the actin cytoskeleton leading to disruption of the endothelial cell monolayer, intercellular gap formation and increased permeability of the monolayer, which did not occur upon heme exposure (Silva et al., 2009). Both heme and oxHb have been shown to induce inflammation in mice, with the notion that oxHb seems to be a 10-times more potent agonist than heme (Wagener et al., 2001; Silva et al., 2009). Both heme and oxHb are chemotactic for neutrophils when injected into the peritoneal cavity of mice, but again, oxHb is a much stronger chemotactic agent compared to heme (Porto et al., 2007; Silva et al., 2009). Importantly, heme and oxHb-mediated inflammatory responses do not share a common signaling pathway, as heme mediated response has been shown to be TLR4-depenedent (Figueiredo et al., 2007; Belcher et al., 2014), whereas oxHb acts on a TLR4-independent manner (Silva et al., 2009).

Macrophages are considered to be the major immune cell type involved in atherogenesis. These macrophages originate from blood monocytes which are attracted to the subendothelial space. The plaque microenvironment dictates the differentiation of these cells functionally diverse phenotypes. Besides the most extensively studied M1 and M2 subtypes, several other macrophage populations have been identified in atherosclerotic plaques (reviewed in Leitinger and Schulman, 2013; Vinchi et al., 2014). Boyle et al. recently identified a novel hemorrhage-associated macrophage phenotype (Mhem, HA-mac) in human hemorrhaged atherosclerotic plaques (Boyle et al., 2009). It has been demonstrated that polarization of these Mhem macrophages is driven by Hb bound to its endogenous scavenger Hp (Boyle et al., 2009; Finn et al., 2012). The major function of Mhem macrophages is the safe elimination of cell free Hb from the plaque, therefore they highly express CD163, the receptor for uptake of Hb:Hp complex and HO-1, the rate limiting enzyme of heme catabolism (Boyle et al., 2009, 2012). Moreover, Mhem differentiation prevents foam cell formation via decreased lipid uptake and increased cholesterol efflux (Finn et al., 2012). All of these properties can contribute to the atheroprotective nature of these Mhem macrophages (Figure 1).

### Defense and adaptation mechanisms

Extracellular Hb and heme are harmful therefore efficient mechanisms have evolved to control their deleterious effects (Figure 1). The plasma acute phase proteins Hp and Hx are in the first line of defense upon intravascular hemolysis. The protective strategy is completed with the HO-1/ferritin system that could serve as the last line of defense and become activated when the Hp and Hx cannot control free Hb and heme mediated stress (Figure 1). The pharmacological potential of these molecules emerged recently to neutralize the adverse effects of Hb and heme in diverse pathologies (Durante, 2010; Schaer et al., 2013a).

#### Control of free Hb by Hp

Hp is present in plasma in high amounts (0.41–1.65 mg/ml) with the exclusive recognized function of capturing and chaperoning cell free Hb to macrophages for degradation (Figure 1) (reviewed in Alayash, 2011). Hp binding accelerates the elimination of circulating Hb through the CD163 macrophage scavenger receptor-mediated endocytosis (Kristiansen et al., 2001). The Hp:Hb complex is highly stable and protects Hb from H_2_O_2_-induced oxidation (Miller et al., 1997; Buehler et al., 2009; Pimenova et al., 2010; Banerjee et al., 2012; Potor et al., 2013; Schaer et al., 2013b). Recent resolution of the crystal structure of the porcine Hp:Hb complex revealed that Hb residues known to be prone to oxidative modifications are buried in the Hp:Hb interface thereby explaining the protective effect of Hp against H_2_O_2_-induced oxidation (Andersen et al., 2012). Hp binding not just provide structural stabilization of Hb but also inhibits heme transfer from Hb toward LDL or vascular endothelial cells (Balla et al., 1993; Nagy et al., 2010; Schaer et al., 2013b).

In humans there are two alleles for the Hp gene resulting 3 different genotypes Hp1-1, Hp2-1 and Hp2-2 (reviewed in Goldenstein et al., 2012) accompanied by structurally different proteins. This molecular heterogeneity of Hp was found to be associated with cardiovascular diseases. Many clinical observations revealed that the Hp2-2 genotype is a risk factor for cardiovascular complications in diverse patient populations (reviewed in Costacou and Levy, 2012), however the attempt to understand the underlying mechanisms lead to controversial results. It has been demonstrated that Hp1-1 is more efficient in blocking heme transfer from Hb to LDL or endothelial cells than Hp2-2 (Melamed-Frank et al., 2001; Bamm et al., 2004) but recently it was reported that the two proteins are equally efficient (Lipiski et al., 2013). Furthermore, Hp2-2:Hb complex was found to be associated with higher functional affinity for the macrophage scavenger receptor CD163 than the Hp1-1:Hb complex (Kristiansen et al., 2001), though other group observed the opposite (Asleh et al., 2003).

Nevertheless, the protective effect and the therapeutic potential of Hp in various hemolytic models has been reported (reviewed in Schaer et al., 2013a), but whether Hb scavenging by Hp acts in an atheroprotective manner remained to be elucidated.

#### Control of free hem by Hx

Upon excessive hemolysis Hp is consumed, causing accumulation and oxidation of cell-free Hb that eventually lead to the release of heme. Hx is an acute-phase plasma protein that binds heme with the highest affinity of any known heme-binding proteins (Hrkal et al., 1974). Hx-heme complexes are internalized via the scavenger receptor LDL receptor-related protein 1/CD91 (Hvidberg et al., 2005) mainly by hepatocytes and macrophages (Figure 1) (Herz and Strickland, 2001). Following endocytosis heme is degraded by HO-1 and iron is stored by ferritin (Alam and Smith, 1989). Although it is well-established that Hx binding inhibits the catalytic activity of heme in oxidative reactions including LDL oxidation (Figure 1) (Gutteridge and Smith, 1988; Vincent et al., 1988; Balla et al., 1991a), its role in atherogenesis remained to be elucidated.

Larsen et al. showed that Hx can be used therapeutically to attenuate heme-mediated tissue damage upon severe sepsis in mice (Larsen et al., 2010). Following this study the protective nature of Hx has been shown in different hemolytic mice models, in which administration of Hx improved cardiac function of those mice (Vinchi et al., 2013). These studies raise the possibility of Hx-based therapeutics in the treatment of diverse pathologies in which heme-mediated tissue damage play an etiopathogenetic role.

#### The HO-1 ferritin system

Cells exposed to free heme or heme-releasing Hb species, i.e., metHb and oxHb up-regulate HO-1 and ferritin (Figure 1) (Balla et al., 1992a,b; Nath et al., 1992; Potor et al., 2013). These proteins provide cellular and tissue protection in diverse pathologies. The mechanism of cytoprotection by HO-1 was recently reviewed (Gozzelino et al., 2010), and it relies on the ability of HO-1 to degrade heme into biliverdin—that is promptly converted into biliverdin—carbon-monoxide (CO) and iron (Tenhunen et al., 1968). The subsequent upregulation of ferritin is essential to obtain the protective effect, as it can store the released iron in a catalitically inactive form (Balla et al., 1992b). Additionally, the side-products of heme degradation—bilirubin and CO—exert various antioxidant and anti-inflammatory properties (Gozzelino et al., 2010).

Many lines of evidences support the atheroprotective nature of HO-1 in humans and in experimental atherosclerosis (reviewed in Vinchi et al., 2014). Recently it has been shown that HO-1 not simply slow down, but reverse plaque progression from a vulnerable plaque to a more stable phenotype (Cheng et al., 2009).

Autopsy examinations of human atherosclerotic lesions revealed that HO-1 expression correlates with plaque instability and the level of pro-inflammatory markers. This might be explained by the stress responsive nature of HO-1. Initial induction of HO-1 expression may act as a compensatory atheroprotective mechanism, whereas at later stages HO-1 expression reflects oxidative stress, inflammation and tissue damage.

Upregulation of HO-1 in atherosclerotic lesions generally appears to coincide with ferritin induction *in vitro* (Juckett et al., 1995; Pang et al., 1996). The increased expression of ferritin also reflects cellular response to heme or heme-iron generated lipid peroxidation products (Agarwal et al., 1996; Hill-Kapturczak et al., 2003). Such induction correlates with the oxidative insult imposed by reactive oxygen and iron. The mechanism by which ferritin provides cytoprotection relies on the ferroxidase activity of H-ferritin subunit (Balla et al., 1992b). Beyond cytoprotection, ferritin serves as a regulator for cell proliferation, inflammation and vascular calcification (Figure 1) (reviewed in Crawford and Blankenhorn, 1991; Zarjou et al., 2009).

#### Impaired defense mechanisms in the atherosclerotic lesion

The elimination of cell-free Hb and heme by Hp and Hx is well characterized in hemolytic pathologies where Hb is released into the circulation. But our knowledge is quite limited when Hb is released from RBC outside of the circulatory system. Hp and Hx are plasma proteins and their penetration into the deeper compartments of atherosclerosic plaque might be limited. This could be particularly true for Hp2-2 that is a large molecule thereby its restricted diffusion may explain the apparent association of the Hp2-2 genotype with more severe symptoms in different pathologies.

Following IPH oxidation of Hb occurs, leading to the formation of structurally altered (e.g., covalently cross-linked) Hb species. It was hypothesized that these structural changes might be associated with the impairment of the endogenous scavenging pathways. Recent studies have revealed that elimination of oxidized Hb species via both high-affinity and low-affinity pathways are severely compromised (Schaer et al., 2006; Vallelian et al., 2008).

Impaired defense mechanisms following IPH might limit the clearance of extracellular Hb and heme from the atherosclerotic plaque thereby this could be a new etiopathogenic factor to address in details in the future.

#### Control of free heme in extravascular sites by α-1 microglobulin (A1M)

A1M is a small glycoprotein that is found ubiquitously in all tissues. Recently it has been demonstrated that A1M can bind small molecules in its hydrophobic pocket, scavenge free radicals and possesses reductase activity. Based on these features A1M plays a crucial role in tissue housekeeping (reviewed in Akerstrom and Gram, 2014). Importantly, heme is a major ligand for A1M that can bind heme with high affinity and degrade it (Allhorn et al., 2002). The protective effect of A1M against Hb/heme-mediated oxidative stress has been shown in different *in vitro* models (Olsson et al., 2008, 2011). Moreover, recently it was demonstrated that A1M infusion attenuates Hb-induced kidney damage in rats (Sverrisson et al., 2014). Based on these properties, we can assume that A1M plays a beneficial role upon IPH by neutralizing and eliminating radicals, oxidants and free heme, but this hypothesis and the potential therapeutic potential of A1M needs to be tested in the future.

## Conclusions

In the last decade, our understanding of atherosclerotic plaque progression and vulnerability underwent a fundamental revision, and neovascularization accompanied by IPH shifted from being an innocent bystander to a pathogenic event that plays a critical role in atherogenesis.

Extravasation of RBCs into the plaque is of crucial importance in triggering IPH-associated reactions. RBC membrane lipids contribute to plaque expansion, whereas cell-free Hb and its oxidation products are strong pro-oxidants and pro-inflammatory agonists targeting cell types with major roles in atherogenesis, such as vascular endothelial cells and macrophages. Systemic and cellular defense strategies to cope with extracellular Hb and its oxidation products might not be efficient or sufficient enough to control the deleterious effects of these molecules deep inside the atherosclerotic plaque, the “death zone.”

Comprehensive understanding the role of neovascularization, IPH and Hb release and oxidation on atherogenesis may lead to the development of novel therapeutics intended to interrupt these pathological events.

### Conflict of interest statement

The authors declare that the research was conducted in the absence of any commercial or financial relationships that could be construed as a potential conflict of interest.

## References

[B1] Agarwal A. Balla J. Balla G. Croatt A. J. Vercellotti G. M. Nath K. A. (1996). Renal tubular epithelial cells mimic endothelial cells upon exposure to oxidized LDL. Am. J. Physiol. 271, F814–F823 8898011 10.1152/ajprenal.1996.271.4.F814

[B2] Akerstrom B. Gram M. (2014). A1M, an extravascular tissue cleaning and housekeeping protein. Free Radic. Biol. Med. 74C, 274–282 10.1016/j.freeradbiomed.2014.06.025 25035076

[B3] Alam J. Smith A. (1989). Receptor-mediated transport of heme by hemopexin regulates gene expression in mammalian cells. J. Biol. Chem. 264, 17637–17640 2553689

[B4] Alayash A. I. (2011). Haptoglobin: Old protein with new functions. Clin. Chim. Acta 412, 493–498 10.1016/j.cca.2010.12.011 21159311

[B5] Alayash A. I. Patel R. P. Cashon R. E. (2001). Redox reactions of hemoglobin and myoglobin: biological and toxicological implications. Antioxid. Redox Signal. 3, 313–327 10.1089/152308601300185250 11396484

[B6] Allhorn M. Berggard T. Nordberg J. Olsson M. L. Akerstrom B. (2002). Processing of the lipocalin alpha(1)-microglobulin by hemoglobin induces heme-binding and heme-degradation properties. Blood 99, 1894–1901 10.1182/blood.V99.6.1894 11877257

[B7] Andersen C. B. Torvund-Jensen M. Nielsen M. J. de Oliveira C. L. Hersleth H. P. Andersen N. H. (2012). Structure of the haptoglobin-haemoglobin complex. Nature 489, 456–459 10.1038/nature11369 22922649

[B8] Arruda M. A. Graca-Souza A. V. Barja-Fidalgo C. (2005). Heme and innate immunity: new insights for an old molecule. Mem. Inst. Oswaldo Cruz 100, 799–803 10.1590/S0074-02762005000700022 16410972

[B9] Asleh R. Marsh S. Shilkrut M. Binah O. Guetta J. Lejbkowicz F. (2003). Genetically determined heterogeneity in hemoglobin scavenging and susceptibility to diabetic cardiovascular disease. Circ. Res. 92, 1193–1200 10.1161/01.RES.0000076889.23082.F1 12750308

[B10] Balla G. Jacob H. S. Balla J. Rosenberg M. Nath K. Apple F. (1992b). Ferritin: a cytoprotective antioxidant strategem of endothelium. J. Biol. Chem. 267, 18148–18153 1517245

[B11] Balla G. Jacob H. S. Eaton J. W. Belcher J. D. Vercellotti G. M. (1991a). Hemin: a possible physiological mediator of low density lipoprotein oxidation and endothelial injury. Arterioscler. Thromb. 11, 1700–1711 1931871 10.1161/01.atv.11.6.1700

[B12] Balla G. Vercellotti G. Eaton J. W. Jacob H. S. (1990). Heme uptake by endothelium synergizes polymorphonuclear granulocyte-mediated damage. Trans. Assoc. Am. Physicians 103, 174–179 2132529

[B13] Balla G. Vercellotti G. M. Muller-Eberhard U. Eaton J. Jacob H. S. (1991b). Exposure of endothelial cells to free heme potentiates damage mediated by granulocytes and toxic oxygen species. Lab. Invest. 64, 648–655 2030579

[B14] Balla J. Jacob H. S. Balla G. Nath K. Eaton J. W. Vercellotti G. M. (1993). Endothelial-cell heme uptake from heme proteins: induction of sensitization and desensitization to oxidant damage. Proc. Natl. Acad. Sci. U.S.A. 90, 9285–9289 10.1073/pnas.90.20.9285 8415693 PMC47552

[B15] Balla J. Jacob H. S. Balla G. Nath K. Vercellotti G. M. (1992a). Endothelial cell heme oxygenase and ferritin induction by heme proteins: a possible mechanism limiting shock damage. Trans. Assoc. Am. Physicians 105, 1–6 1308986

[B16] Balla J. Vercellotti G. M. Jeney V. Yachie A. Varga Z. Jacob H. S. (2007). Heme, heme oxygenase, and ferritin: how the vascular endothelium survives (and dies) in an iron-rich environment. Antioxid. Redox Signal. 9, 2119–2137 10.1089/ars.2007.1787 17767398

[B17] Bamm V. V. Tsemakhovich V. A. Shaklai M. Shaklai N. (2004). Haptoglobin phenotypes differ in their ability to inhibit heme transfer from hemoglobin to LDL. Biochemistry 43, 3899–3906 10.1021/bi0362626 15049697

[B18] Banerjee S. Jia Y. Siburt C. J. Abraham B. Wood F. Bonaventura C. (2012). Haptoglobin alters oxygenation and oxidation of hemoglobin and decreases propagation of peroxide-induced oxidative reactions. Free Radic. Biol. Med. 53, 1317–1326 10.1016/j.freeradbiomed.2012.07.023 22841869

[B19] Belcher J. D. Chen C. Nguyen J. Milbauer L. Abdulla F. Alayash A. I. (2014). Heme triggers TLR4 signaling leading to endothelial cell activation and vaso-occlusion in murine sickle cell disease. Blood 123, 377–390 10.1182/blood-2013-04-495887 24277079 PMC3894494

[B20] Bhandaru R. Srinivasan S. R. Radhakrisnamurthy B. Berenson G. S. (1982). Effects of diabetes and high fat-high cholesterol diet on plasma lipid levels and on erythrocyte membrane composition. Atherosclerosis 42, 263–272 10.1016/0021-9150(82)90156-3 6462153

[B21] Bjornheden T. Levin M. Evaldsson M. Wiklund O. (1999). Evidence of hypoxic areas within the arterial wall *in vivo*. Arterioscler. Thromb. Vasc. Biol. 19, 870–876 10.1161/01.ATV.19.4.870 10195911

[B22] Bochkov V. N. Philippova M. Oskolkova O. Kadl A. Furnkranz A. Karabeg E. (2006). Oxidized phospholipids stimulate angiogenesis via autocrine mechanisms, implicating a novel role for lipid oxidation in the evolution of atherosclerotic lesions. Circ. Res. 99, 900–908 10.1161/01.RES.0000245485.04489.ee 16973904

[B23] Bordon Y. (2010). A new vein of TLR biology. Nat. Rev. Immunol. 10:748 10.1038/nri2875 21080611

[B24] Bot I. Jukema J. W. Lankhuizen I. M. van Berkel T. J. Biessen E. A. (2011). Atorvastatin inhibits plaque development and adventitial neovascularization in ApoE deficient mice independent of plasma cholesterol levels. Atherosclerosis 214, 295–300 10.1016/j.atherosclerosis.2010.11.008 21130458

[B25] Boyle J. J. Harrington H. A. Piper E. Elderfield K. Stark J. Landis R. C. (2009). Coronary intraplaque hemorrhage evokes a novel atheroprotective macrophage phenotype. Am. J. Pathol. 174, 1097–1108 10.2353/ajpath.2009.080431 19234137 PMC2665768

[B26] Boyle J. J. Johns M. Kampfer T. Nguyen A. T. Game L. Schaer D. J. (2012). Activating transcription factor 1 directs Mhem atheroprotective macrophages through coordinated iron handling and foam cell protection. Circ. Res. 110, 20–33 10.1161/CIRCRESAHA.111.247577 22052915

[B27] Buehler P. W. Abraham B. Vallelian F. Linnemayr C. Pereira C. P. Cipollo J. F. (2009). Haptoglobin preserves the CD163 hemoglobin scavenger pathway by shielding hemoglobin from peroxidative modification. Blood 113, 2578–2586 10.1182/blood-2008-08-174466 19131549

[B28] Bunn H. F. Jandl J. H. (1968). Exchange of heme among hemoglobins and between hemoglobin and albumin. J. Biol. Chem. 243, 465–475 4966113

[B29] Caspar-Bauguil S. Garcia J. Galinier A. Periquet B. Ferrieres J. Allenbach S. (2010). Positive impact of long-term lifestyle change on erythrocyte fatty acid profile after acute coronary syndromes. Arch. Cardiovasc. Dis. 103, 106–114 10.1016/j.acvd.2009.12.005 20226430

[B30] Celletti F. L. Waugh J. M. Amabile P. G. Brendolan A. Hilfiker P. R. Dake M. D. (2001). Vascular endothelial growth factor enhances atherosclerotic plaque progression. Nat. Med. 7, 425–429 10.1038/86490 11283668

[B31] Cheng C. Noordeloos A. M. Jeney V. Soares M. P. Moll F. Pasterkamp G. (2009). Heme oxygenase 1 determines atherosclerotic lesion progression into a vulnerable plaque. Circulation 119, 3017–3027 10.1161/CIRCULATIONAHA.108.808618 19487598

[B32] Costacou T. Levy A. P. (2012). Haptoglobin genotype and its role in diabetic cardiovascular disease. J. Cardiovasc. Transl. Res. 5, 423–435 10.1007/s12265-012-9361-z 22447230 PMC3595557

[B33] Crawford D. W. Blankenhorn D. H. (1991). Arterial wall oxygenation, oxyradicals, and atherosclerosis. Atherosclerosis 89, 97–108 10.1016/0021-9150(91)90049-9 1793456

[B34] Deterding L. J. Ramirez D. C. Dubin J. R. Mason R. P. Tomer K. B. (2004). Identification of free radicals on hemoglobin from its self-peroxidation using mass spectrometry and immuno-spin trapping: observation of a histidinyl radical. J. Biol. Chem. 279, 11600–11607 10.1074/jbc.M310704200 14699100

[B35] Drinane M. Mollmark J. Zagorchev L. Moodie K. Sun B. Hall A. (2009). The antiangiogenic activity of rPAI-1(23) inhibits vasa vasorum and growth of atherosclerotic plaque. Circ. Res. 104, 337–345 10.1161/CIRCRESAHA.108.184622 19122176 PMC2737332

[B36] Dunmore B. J. McCarthy M. J. Naylor A. R. Brindle N. P. (2007). Carotid plaque instability and ischemic symptoms are linked to immaturity of microvessels within plaques. J. Vasc. Surg. 45, 155–159 10.1016/j.jvs.2006.08.072 17210401

[B37] Durante W. (2010). Targeting heme oxygenase-1 in vascular disease. Curr. Drug Targets 11, 1504–1516 10.2174/1389450111009011504 20704550 PMC2978667

[B38] Figueiredo R. T. Fernandez P. L. Mourao-Sa D. S. Porto B. N. Dutra F. F. Alves L. S. (2007). Characterization of heme as activator of Toll-like receptor 4. J. Biol. Chem. 282, 20221–20229 10.1074/jbc.M610737200 17502383

[B39] Finn A. V. Nakano M. Polavarapu R. Karmali V. Saeed O. Zhao X. (2012). Hemoglobin directs macrophage differentiation and prevents foam cell formation in human atherosclerotic plaques. J. Am. Coll. Cardiol. 59, 166–177 10.1016/j.jacc.2011.10.852 22154776 PMC3253238

[B40] Forsythe J. A. Jiang B. H. Iyer N. V. Agani F. Leung S. W. Koos R. D. (1996). Activation of vascular endothelial growth factor gene transcription by hypoxia-inducible factor 1. Mol. Cell. Biol. 16, 4604–4613 8756616 10.1128/mcb.16.9.4604 PMC231459

[B41] Galis Z. S. Lessner S. M. (2009). Will the real plaque vasculature please stand up? Why we need to distinguish the vasa plaquorum from the vasa vasorum. Trends Cardiovasc. Med. 19, 87–94 10.1016/j.tcm.2009.06.001 19679265 PMC6620604

[B42] Gao L. Chen Q. Zhou X. Fan L. (2012). The role of hypoxia-inducible factor 1 in atherosclerosis. J. Clin. Pathol. 65, 872–876 10.1136/jclinpath-2012-200828 22569539

[B43] Geiringer E. (1951). Intimal vascularization and atherosclerosis. J. Pathol. Bacteriol. 63, 201–211 10.1002/path.1700630204 14851161

[B44] Goldenstein H. Levy N. S. Levy A. P. (2012). Haptoglobin genotype and its role in determining heme-iron mediated vascular disease. Pharmacol. Res. 66, 1–6 10.1016/j.phrs.2012.02.011 22465143 PMC3345090

[B45] Gozzelino R. Jeney V. Soares M. P. (2010). Mechanisms of cell protection by heme oxygenase-1. Annu. Rev. Pharmacol. Toxicol. 50, 323–354 10.1146/annurev.pharmtox.010909.105600 20055707

[B46] Gram M. Sveinsdottir S. Ruscher K. Hansson S. R. Cinthio M. Akerstrom B. (2013). Hemoglobin induces inflammation after preterm intraventricular hemorrhage by methemoglobin formation. J. Neuroinflammation 10:100 10.1186/1742-2094-10-100 23915174 PMC3750409

[B47] Grote K. Schuett H. Salguero G. Grothusen C. Jagielska J. Drexler H. (2010). Toll-like receptor 2/6 stimulation promotes angiogenesis via GM-CSF as a potential strategy for immune defense and tissue regeneration. Blood 115, 2543–2552 10.1182/blood-2009-05-224402 20056792

[B48] Gutteridge J. M. Smith A. (1988). Antioxidant protection by haemopexin of haem-stimulated lipid peroxidation. Biochem. J. 256, 861–865 3223958 10.1042/bj2560861 PMC1135495

[B49] Harel S. Kanner J. (1988). The generation of ferryl or hydroxyl radicals during interaction of haemproteins with hydrogen peroxide. Free Radic. Res. Commun. 5, 21–33 10.3109/10715768809068555 2853114

[B50] Heeschen C. Jang J. J. Weis M. Pathak A. Kaji S. Hu R. S. (2001). Nicotine stimulates angiogenesis and promotes tumor growth and atherosclerosis. Nat. Med. 7, 833–839 10.1038/89961 11433349

[B51] Heistad D. D. Armstrong M. L. Marcus M. L. (1981). Hyperemia of the aortic wall in atherosclerotic monkeys. Circ. Res. 48, 669–675 10.1161/01.RES.48.5.669 7214675

[B52] Herz J. Strickland D. K. (2001). LRP: a multifunctional scavenger and signaling receptor. J. Clin. Invest. 108, 779–784 10.1172/JCI200113992 11560943 PMC200939

[B53] Higashida T. Kanno H. Nakano M. Funakoshi K. Yamamoto I. (2008). Expression of hypoxia-inducible angiogenic proteins (hypoxia-inducible factor-1alpha, vascular endothelial growth factor, and E26 transformation-specific-1) and plaque hemorrhage in human carotid atherosclerosis. J. Neurosurg. 109, 83–91 10.3171/JNS/2008/109/7/0083 18590436

[B54] Hill-Kapturczak N. Voakes C. Garcia J. Visner G. Nick H. S. Agarwal A. (2003). A cis-acting region regulates oxidized lipid-mediated induction of the human heme oxygenase-1 gene in endothelial cells. Arterioscler. Thromb. Vasc. Biol. 23, 1416–1422 10.1161/01.ATV.0000081656.76378.A7 12805077

[B55] Hrkal Z. Vodrazka Z. Kalousek I. (1974). Transfer of heme from ferrihemoglobin and ferrihemoglobin isolated chains to hemopexin. Eur. J. Biochem. 43, 73–78 10.1111/j.1432-1033.1974.tb03386.x 4209590

[B56] Hutter R. Speidl W. S. Valdiviezo C. Sauter B. Corti R. Fuster V. (2013). Macrophages transmit potent proangiogenic effects of oxLDL *in vitro* and *in vivo* involving HIF-1alpha activation: a novel aspect of angiogenesis in atherosclerosis. J. Cardiovasc. Transl. Res. 6, 558–569 10.1007/s12265-013-9469-9 23661177 PMC4334467

[B57] Hvidberg V. Maniecki M. B. Jacobsen C. Hojrup P. Moller H. J. Moestrup S. K. (2005). Identification of the receptor scavenging hemopexin-heme complexes. Blood 106, 2572–2579 10.1182/blood-2005-03-1185 15947085

[B58] Ivan M. Kondo K. Yang H. Kim W. Valiando J. Ohh M. (2001). HIFalpha targeted for VHL-mediated destruction by proline hydroxylation: implications for O2 sensing. Science 292, 464–468 10.1126/science.1059817 11292862

[B59] Ivanov A. S. Torkhovskaya T. I. Khalilov E. M. Archakov A. I. (1991). Diet-induced hypercholesterolaemia in the rabbit. Biomed. Sci. 2, 285–288 1751762

[B60] Jeney V. Balla J. Yachie A. Varga Z. Vercellotti G. M. Eaton J. W. (2002). Pro-oxidant and cytotoxic effects of circulating heme. Blood 100, 879–887 10.1182/blood.V100.3.879 12130498

[B61] Jeney V. Eaton J. W. Balla G. Balla J. (2013). Natural history of the bruise: formation, elimination, and biological effects of oxidized hemoglobin. Oxid. Med. Cell. Longev. 2013:703571 10.1155/2013/703571 23766858 PMC3671564

[B62] Jeziorska M. Woolley D. E. (1999). Neovascularization in early atherosclerotic lesions of human carotid arteries: its potential contribution to plaque development. Hum. Pathol. 30, 919–925 10.1016/S0046-8177(99)90245-9 10452504

[B63] Jia Y. Buehler P. W. Boykins R. A. Venable R. M. Alayash A. I. (2007). Structural basis of peroxide-mediated changes in human hemoglobin: a novel oxidative pathway. J. Biol. Chem. 282, 4894–4907 10.1074/jbc.M609955200 17178725

[B64] Juan S. H. Lee T. S. Tseng K. W. Liou J. Y. Shyue S. K. Wu K. K. (2001). Adenovirus-mediated heme oxygenase-1 gene transfer inhibits the development of atherosclerosis in apolipoprotein E-deficient mice. Circulation 104, 1519–1525 10.1161/hc3801.095663 11571246

[B65] Juckett M. B. Balla J. Balla G. Jessurun J. Jacob H. S. Vercellotti G. M. (1995). Ferritin protects endothelial cells from oxidized low density lipoprotein *in vitro*. Am. J. Pathol. 147, 782–789 7677189 PMC1870976

[B66] Julius U. Pietzsch J. (2005). Glucose-induced enhancement of hemin-catalyzed LDL oxidation *in vitro* and *in vivo*. Antioxid. Redox Signal. 7, 1507–1512 10.1089/ars.2005.7.1507 16356114

[B67] Jun J. Reinke C. Bedja D. Berkowitz D. Bevans-Fonti S. Li J. (2010). Effect of intermittent hypoxia on atherosclerosis in apolipoprotein E-deficient mice. Atherosclerosis 209, 381–386 10.1016/j.atherosclerosis.2009.10.017 19897196 PMC2846209

[B68] Jurrus E. R. Weiss H. S. (1977). *In vitro* tissue oxygen tensions in the rabbit aortic arch. Atherosclerosis 28, 223–232 10.1016/0021-9150(77)90172-1 597341

[B69] Kawashima A. Oda Y. Yachie A. Koizumi S. Nakanishi I. (2002). Heme oxygenase-1 deficiency: the first autopsy case. Hum. Pathol. 33, 125–130 10.1053/hupa.2002.30217 11823983

[B70] Kim Y. W. Byzova T. V. (2014). Oxidative stress in angiogenesis and vascular disease. Blood 123, 625–631 10.1182/blood-2013-09-512749 24300855 PMC3907751

[B71] Knighton D. R. Hunt T. K. Scheuenstuhl H. Halliday B. J. Werb Z. Banda M. J. (1983). Oxygen tension regulates the expression of angiogenesis factor by macrophages. Science 221, 1283–1285 10.1126/science.6612342 6612342

[B72] Kockx M. M. Cromheeke K. M. Knaapen M. W. Bosmans J. M. De Meyer G. R. Herman A. G. (2003). Phagocytosis and macrophage activation associated with hemorrhagic microvessels in human atherosclerosis. Arterioscler. Thromb. Vasc. Biol. 23, 440–446 10.1161/01.ATV.0000057807.28754.7F 12615689

[B73] Kolodgie F. D. Burke A. P. Nakazawa G. Cheng Q. Xu X. Virmani R. (2007). Free cholesterol in atherosclerotic plaques: where does it come from? Curr. Opin. Lipidol. 18, 500–507 10.1097/MOL.0b013e3282efa35b 17885419

[B74] Kolodgie F. D. Gold H. K. Burke A. P. Fowler D. R. Kruth H. S. Weber D. K. (2003). Intraplaque hemorrhage and progression of coronary atheroma. N. Engl. J. Med. 349, 2316–2325 10.1056/NEJMoa035655 14668457

[B75] Koter M. Broncel M. Chojnowska-Jezierska J. Klikczynska K. Franiak I. (2002). The effect of atorvastatin on erythrocyte membranes and serum lipids in patients with type-2 hypercholesterolemia. Eur. J. Clin. Pharmacol. 58, 501–506 10.1007/s00228-002-0507-9 12451426

[B76] Kristiansen M. Graversen J. H. Jacobsen C. Sonne O. Hoffman H. J. Law S. K. (2001). Identification of the haemoglobin scavenger receptor. Nature 409, 198–201 10.1038/35051594 11196644

[B77] Larsen R. Gozzelino R. Jeney V. Tokaji L. Bozza F. A. Japiassu A. M. (2010). A central role for free heme in the pathogenesis of severe sepsis. Sci. Transl. Med. 2:51ra71 10.1126/scitranslmed.3001118 20881280

[B78] Leibovich S. J. Chen J. F. Pinhal-Enfield G. Belem P. C. Elson G. Rosania A. (2002). Synergistic up-regulation of vascular endothelial growth factor expression in murine macrophages by adenosine A(2A) receptor agonists and endotoxin. Am. J. Pathol. 160, 2231–2244 10.1016/S0002-9440(10)61170-4 12057925 PMC1850844

[B79] Leitinger N. Schulman I. G. (2013). Phenotypic polarization of macrophages in atherosclerosis. Arterioscler. Thromb. Vasc. Biol. 33, 1120–1126 10.1161/ATVBAHA.112.300173 23640492 PMC3745999

[B80] Leppanen O. Bjornheden T. Evaldsson M. Boren J. Wiklund O. Levin M. (2006). ATP depletion in macrophages in the core of advanced rabbit atherosclerotic plaques *in vivo*. Atherosclerosis 188, 323–330 10.1016/j.atherosclerosis.2005.11.017 16405894

[B81] Levin M. Leppanen O. Evaldsson M. Wiklund O. Bondjers G. Bjornheden T. (2003). Mapping of ATP, glucose, glycogen, and lactate concentrations within the arterial wall. Arterioscler. Thromb. Vasc. Biol. 23, 1801–1807 10.1161/01.ATV.0000092872.54026.8D 12947013

[B82] Li W. Ostblom M. Xu L. H. Hellsten A. Leanderson P. Liedberg B. (2006). Cytocidal effects of atheromatous plaque components: the death zone revisited. FASEB J. 20, 2281–2290 10.1096/fj.06-6114com 17077305

[B83] Libby P. (2002). Inflammation in atherosclerosis. Nature 420, 868–874 10.1038/nature01323 12490960

[B84] Lipiski M. Deuel J. Baek J. H. Engelsberger W. R. Buehler P. W. Schaer D. J. (2013). Human phenotype specific haptoglobin therapeutics are both effective *in vitro* and *in vivo* to attenuate hemoglobin toxicity in guinea pigs. Antioxid. Redox Signal. 10.1089/ars.2012.5089 23418677 PMC3809386

[B85] Lusis A. J. (2000). Atherosclerosis. Nature 407, 233–241 10.1038/35025203 11001066 PMC2826222

[B86] Luttun A. Tjwa M. Moons L. Wu Y. Angelillo-Scherrer A. Liao F. (2002). Revascularization of ischemic tissues by PlGF treatment, and inhibition of tumor angiogenesis, arthritis and atherosclerosis by anti-Flt1. Nat. Med. 8, 831–840 10.1038/nm731 12091877

[B87] Matzinger P. (1994). Tolerance, danger, and the extended family. Annu. Rev. Immunol. 12, 991–1045 10.1146/annurev.iy.12.040194.005015 8011301

[B88] Matzinger P. (2002). The danger model: a renewed sense of self. Science 296, 301–305 10.1126/science.1071059 11951032

[B89] Maxwell P. H. Wiesener M. S. Chang G. W. Clifford S. C. Vaux E. C. Cockman M. E. (1999). The tumour suppressor protein VHL targets hypoxia-inducible factors for oxygen-dependent proteolysis. Nature 399, 271–275 10.1038/20459 10353251

[B90] McCarthy M. J. Loftus I. M. Thompson M. M. Jones L. London N. J. Bell P. R. (1999). Angiogenesis and the atherosclerotic carotid plaque: an association between symptomatology and plaque morphology. J. Vasc. Surg. 30, 261–268 10.1016/S0741-5214(99)70136-9 10436445

[B91] Melamed-Frank M. Lache O. Enav B. I. Szafranek T. Levy N. S. Ricklis R. M. (2001). Structure-function analysis of the antioxidant properties of haptoglobin. Blood 98, 3693–3698 10.1182/blood.V98.13.3693 11739174

[B92] Michel J. B. Virmani R. Arbustini E. Pasterkamp G. (2011). Intraplaque haemorrhages as the trigger of plaque vulnerability. Eur. Heart J. 32, 1977–1985, 1985a, 1985b, 1985c. 10.1093/eurheartj/ehr054 21398643 PMC3155759

[B93] Miller Y. I. Altamentova S. M. Shaklai N. (1997). Oxidation of low-density lipoprotein by hemoglobin stems from a heme-initiated globin radical: antioxidant role of haptoglobin. Biochemistry 36, 12189–12198 10.1021/bi970258a 9315856

[B94] Moreno P. R. Purushothaman K. R. Fuster V. Echeverri D. Truszczynska H. Sharma S. K. (2004). Plaque neovascularization is increased in ruptured atherosclerotic lesions of human aorta: implications for plaque vulnerability. Circulation 110, 2032–2038 10.1161/01.CIR.0000143233.87854.23 15451780

[B95] Moreno P. R. Purushothaman K. R. Sirol M. Levy A. P. Fuster V. (2006). Neovascularization in human atherosclerosis. Circulation 113, 2245–2252 10.1161/CIRCULATIONAHA.105.578955 16684874

[B96] Moreno P. R. Purushothaman M. Purushothaman K. R. (2012). Plaque neovascularization: defense mechanisms, betrayal, or a war in progress. Ann. N.Y. Acad. Sci. 1254, 7–17 10.1111/j.1749-6632.2012.06497.x 22548565

[B97] Moulton K. S. Heller E. Konerding M. A. Flynn E. Palinski W. Folkman J. (1999). Angiogenesis inhibitors endostatin or TNP-470 reduce intimal neovascularization and plaque growth in apolipoprotein E-deficient mice. Circulation 99, 1726–1732 10.1161/01.CIR.99.13.1726 10190883

[B98] Moulton K. S. Vakili K. Zurakowski D. Soliman M. Butterfield C. Sylvin E. (2003). Inhibition of plaque neovascularization reduces macrophage accumulation and progression of advanced atherosclerosis. Proc. Natl. Acad. Sci. U.S.A. 100, 4736–4741 10.1073/pnas.0730843100 12682294 PMC153625

[B99] Nagy E. Eaton J. W. Jeney V. Soares M. P. Varga Z. Galajda Z. (2010). Red cells, hemoglobin, heme, iron, and atherogenesis. Arterioscler. Thromb. Vasc. Biol. 30, 1347–1353 10.1161/ATVBAHA.110.206433 20378845 PMC2893144

[B100] Nagy E. Jeney V. Yachie A. Szabo R. P. Wagner O. Vercellotti G. M. (2005). Oxidation of hemoglobin by lipid hydroperoxide associated with low-density lipoprotein (LDL) and increased cytotoxic effect by LDL oxidation in heme oxygenase-1 (HO-1) deficiency. Cell. Mol. Biol. (Noisy-le-grand). 51, 377–385 10.1170/T641 16309588

[B101] Nakano D. Hayashi T. Tazawa N. Yamashita C. Inamoto S. Okuda N. (2005). Chronic hypoxia accelerates the progression of atherosclerosis in apolipoprotein E-knockout mice. Hypertens. Res. 28, 837–845 10.1291/hypres.28.837 16471178

[B102] Nath K. A. Balla G. Vercellotti G. M. Balla J. Jacob H. S. Levitt M. D. (1992). Induction of heme oxygenase is a rapid, protective response in rhabdomyolysis in the rat. J. Clin. Invest. 90, 267–270 10.1172/JCI115847 1634613 PMC443091

[B103] O'Brien E. R. Garvin M. R. Dev R. Stewart D. K. Hinohara T. Simpson J. B. (1994). Angiogenesis in human coronary atherosclerotic plaques. Am. J. Pathol. 145, 883–894 7524331 PMC1887321

[B104] Olsson M. G. Allhorn M. Larsson J. Cederlund M. Lundqvist K. Schmidtchen A. (2011). Up-regulation of A1M/alpha1-microglobulin in skin by heme and reactive oxygen species gives protection from oxidative damage. PLoS ONE 6:e27505 10.1371/journal.pone.0027505 22096585 PMC3214066

[B105] Olsson M. G. Olofsson T. Tapper H. Akerstrom B. (2008). The lipocalin alpha1-microglobulin protects erythroid K562 cells against oxidative damage induced by heme and reactive oxygen species. Free Radic. Res. 42, 725–736 10.1080/10715760802337265 18712632

[B106] Pang J. H. Jiang M. J. Chen Y. L. Wang F. W. Wang D. L. Chu S. H. (1996). Increased ferritin gene expression in atherosclerotic lesions. J. Clin. Invest. 97, 2204–2212 10.1172/JCI118661 8636399 PMC507299

[B107] Paone A. Galli R. Gabellini C. Lukashev D. Starace D. Gorlach A. (2010). Toll-like receptor 3 regulates angiogenesis and apoptosis in prostate cancer cell lines through hypoxia-inducible factor 1 alpha. Neoplasia 12, 539–549 10.1593/neo.92106 20651983 PMC2907580

[B108] Patel R. P. Svistunenko D. A. Darley-Usmar V. M. Symons M. C. Wilson M. T. (1996). Redox cycling of human methaemoglobin by H2O2 yields persistent ferryl iron and protein based radicals. Free Radic. Res. 25, 117–123 10.3109/10715769609149916 8885329

[B109] Petrovan R. J. Kaplan C. D. Reisfeld R. A. Curtiss L. K. (2007). DNA vaccination against VEGF receptor 2 reduces atherosclerosis in LDL receptor-deficient mice. Arterioscler. Thromb. Vasc. Biol. 27, 1095–1100 10.1161/ATVBAHA.106.139246 17303776

[B110] Pimenova T. Pereira C. P. Gehrig P. Buehler P. W. Schaer D. J. Zenobi R. (2010). Quantitative mass spectrometry defines an oxidative hotspot in hemoglobin that is specifically protected by haptoglobin. J. Proteome Res. 9, 4061–4070 10.1021/pr100252e 20568812

[B111] Pollet I. Opina C. J. Zimmerman C. Leong K. G. Wong F. Karsan A. (2003). Bacterial lipopolysaccharide directly induces angiogenesis through TRAF6-mediated activation of NF-kappaB and c-Jun N-terminal kinase. Blood 102, 1740–1742 10.1182/blood-2003-01-0288 12714497

[B112] Porto B. N. Alves L. S. Fernandez P. L. Dutra T. P. Figueiredo R. T. Graca-Souza A. V. (2007). Heme induces neutrophil migration and reactive oxygen species generation through signaling pathways characteristic of chemotactic receptors. J. Biol. Chem. 282, 24430–24436 10.1074/jbc.M703570200 17581818

[B113] Potor L. Banyai E. Becs G. Soares M. P. Balla G. Balla J. (2013). Atherogenesis may involve the prooxidant and proinflammatory effects of ferryl hemoglobin. Oxid. Med. Cell. Longev. 2013:676425 10.1155/2013/676425 23766856 PMC3671302

[B114] Radhakrishnan N. Yadav S. P. Sachdeva A. Pruthi P. K. Sawhney S. Piplani T. (2011). Human heme oxygenase-1 deficiency presenting with hemolysis, nephritis, and asplenia. J. Pediatr. Hematol. Oncol. 33, 74–78 10.1097/MPH.0b013e3181fd2aae 21088618

[B115] Rajamaki K. Lappalainen J. Oorni K. Valimaki E. Matikainen S. Kovanen P. T. (2010). Cholesterol crystals activate the NLRP3 inflammasome in human macrophages: a novel link between cholesterol metabolism and inflammation. PLoS ONE 5:e11765 10.1371/journal.pone.0011765 20668705 PMC2909263

[B116] Ramirez D. C. Chen Y. R. Mason R. P. (2003). Immunochemical detection of hemoglobin-derived radicals formed by reaction with hydrogen peroxide: involvement of a protein-tyrosyl radical. Free Radic. Biol. Med. 34, 830–839 10.1016/S0891-5849(02)01437-5 12654471

[B117] Reeder B. J. Cutruzzola F. Bigotti M. G. Hider R. C. Wilson M. T. (2008). Tyrosine as a redox-active center in electron transfer to ferryl heme in globins. Free Radic. Biol. Med. 44, 274–283 10.1016/j.freeradbiomed.2007.06.030 18215736

[B118] Ritman E. L. Lerman A. (2007). The dynamic vasa vasorum. Cardiovasc. Res. 75, 649–658 10.1016/j.cardiores.2007.06.020 17631284 PMC2121590

[B119] Sahebkar A. Watts G. F. (2013). New LDL-cholesterol lowering therapies: pharmacology, clinical trials, and relevance to acute coronary syndromes. Clin. Ther. 35, 1082–1098 10.1016/j.clinthera.2013.06.019 23932550

[B120] Schaer C. A. Deuel J. W. Bittermann A. G. Rubio I. G. Schoedon G. Spahn D. R. (2013b). Mechanisms of haptoglobin protection against hemoglobin peroxidation triggered endothelial damage. Cell Death Differ. 20, 1569–1579 10.1038/cdd.2013.113 23995229 PMC3792434

[B121] Schaer D. J. Buehler P. W. Alayash A. I. Belcher J. D. Vercellotti G. M. (2013a). Hemolysis and free hemoglobin revisited: exploring hemoglobin and hemin scavengers as a novel class of therapeutic proteins. Blood 121, 1276–1284 10.1182/blood-2012-11-451229 23264591 PMC3578950

[B122] Schaer D. J. Schaer C. A. Buehler P. W. Boykins R. A. Schoedon G. Alayash A. I. (2006). CD163 is the macrophage scavenger receptor for native and chemically modified hemoglobins in the absence of haptoglobin. Blood 107, 373–380 10.1182/blood-2005-03-1014 16189277

[B123] Siems W. G. Sommerburg O. Grune T. (2000). Erythrocyte free radical and energy metabolism. Clin. Nephrol. 53, S9–S17 10746800

[B124] Silva G. Jeney V. Chora A. Larsen R. Balla J. Soares M. P. (2009). Oxidized hemoglobin is an endogenous proinflammatory agonist that targets vascular endothelial cells. J. Biol. Chem. 284, 29582–29595 10.1074/jbc.M109.045344 19700768 PMC2785591

[B125] Sluimer J. C. Gasc J. M. van Wanroij J. L. Kisters N. Groeneweg M. Sollewijn Gelpke M. D. (2008). Hypoxia, hypoxia-inducible transcription factor, and macrophages in human atherosclerotic plaques are correlated with intraplaque angiogenesis. J. Am. Coll. Cardiol. 51, 1258–1265 10.1016/j.jacc.2007.12.025 18371555

[B126] Sluimer J. C. Kolodgie F. D. Bijnens A. P. Maxfield K. Pacheco E. Kutys B. (2009). Thin-walled microvessels in human coronary atherosclerotic plaques show incomplete endothelial junctions relevance of compromised structural integrity for intraplaque microvascular leakage. J. Am. Coll. Cardiol. 53, 1517–1527 10.1016/j.jacc.2008.12.056 19389562 PMC2756458

[B127] Spirig R. Djafarzadeh S. Regueira T. Shaw S. G. von Garnier C. Takala J. (2010). Effects of TLR agonists on the hypoxia-regulated transcription factor HIF-1alpha and dendritic cell maturation under normoxic conditions. PLoS ONE 5:e0010983 10.1371/journal.pone.0010983 20539755 PMC2881864

[B128] Sverrisson K. Axelsson J. Rippe A. Gram M. Akerstrom B. Hansson S. R. (2014). Extracellular fetal hemoglobin induces increases in glomerular permeability: inhibition with alpha1-microglobulin and tempol. Am. J. Physiol. Renal Physiol. 306, F442–F448 10.1152/ajprenal.00502.2013 24338823

[B129] Tenhunen R. Marver H. S. Schmid R. (1968). The enzymatic conversion of heme to bilirubin by microsomal heme oxygenase. Proc. Natl. Acad. Sci. U.S.A. 61, 748–755 10.1073/pnas.61.2.748 4386763 PMC225223

[B130] Torres Filho I. P. Leunig M. Yuan F. Intaglietta M. Jain R. K. (1994). Noninvasive measurement of microvascular and interstitial oxygen profiles in a human tumor in SCID mice. Proc. Natl. Acad. Sci. U.S.A. 91, 2081–2085 10.1073/pnas.91.6.2081 8134352 PMC43313

[B131] Tynan M. B. Nicholls D. P. Maguire S. M. Steele I. C. McMaster C. Moore R. (1995). Erythrocyte membrane fatty acid composition as a marker of dietary compliance in hyperlipidaemic subjects. Atherosclerosis 117, 245–252 10.1016/0021-9150(95)05578-K 8801870

[B132] Tziakas D. Chalikias G. Kapelouzou A. Tentes I. Schafer K. Karayannakos P. (2013). Erythrocyte membrane cholesterol and lipid core growth in a rabbit model of atherosclerosis: modulatory effects of rosuvastatin. Int. J. Cardiol. 170, 173–181 10.1016/j.ijcard.2013.10.070 24215985

[B133] Vallelian F. Pimenova T. Pereira C. P. Abraham B. Mikolajczyk M. G. Schoedon G. (2008). The reaction of hydrogen peroxide with hemoglobin induces extensive alpha-globin crosslinking and impairs the interaction of hemoglobin with endogenous scavenger pathways. Free Radic. Biol. Med. 45, 1150–1158 10.1016/j.freeradbiomed.2008.07.013 18708138

[B134] Vincent S. H. Grady R. W. Shaklai N. Snider J. M. Muller-Eberhard U. (1988). The influence of heme-binding proteins in heme-catalyzed oxidations. Arch. Biochem. Biophys. 265, 539–550 10.1016/0003-9861(88)90159-2 3421724

[B135] Vinchi F. De Franceschi L. Ghigo A. Townes T. Cimino J. Silengo L. (2013). Hemopexin therapy improves cardiovascular function by preventing heme-induced endothelial toxicity in mouse models of hemolytic diseases. Circulation 127, 1317–1329 10.1161/CIRCULATIONAHA.112.130179 23446829

[B136] Vinchi F. Muckenthaler M. U. Da Silva M. C. Balla G. Balla J. Jeney V. (2014). Atherogenesis and iron: from epidemiology to cellular level. Front. Pharmacol. 5:94 10.3389/fphar.2014.00094 24847266 PMC4017151

[B137] Vink A. Schoneveld A. H. Lamers D. Houben A. J. van der Groep P. van Diest P. J. (2007). HIF-1 alpha expression is associated with an atheromatous inflammatory plaque phenotype and upregulated in activated macrophages. Atherosclerosis 195, e69–e75 10.1016/j.atherosclerosis.2007.05.026 17606258

[B138] Wagener F. A. Eggert A. Boerman O. C. Oyen W. J. Verhofstad A. Abraham N. G. (2001). Heme is a potent inducer of inflammation in mice and is counteracted by heme oxygenase. Blood 98, 1802–1811 10.1182/blood.V98.6.1802 11535514

[B139] Wagener F. A. Feldman E. de Witte T. Abraham N. G. (1997). Heme induces the expression of adhesion molecules ICAM-1, VCAM-1, and E selectin in vascular endothelial cells. Proc. Soc. Exp. Biol. Med. 216, 456–463 10.3181/00379727-216-44197 9402154

[B140] Wang G. L. Jiang B. H. Rue E. A. Semenza G. L. (1995). Hypoxia-inducible factor 1 is a basic-helix-loop-helix-PAS heterodimer regulated by cellular O2 tension. Proc. Natl. Acad. Sci. U.S.A. 92, 5510–5514 10.1073/pnas.92.12.5510 7539918 PMC41725

[B141] Wang G. L. Semenza G. L. (1995). Purification and characterization of hypoxia-inducible factor 1. J. Biol. Chem. 270, 1230–1237 10.1074/jbc.270.3.1230 7836384

[B142] Wenger R. H. Stiehl D. P. Camenisch G. (2005). Integration of oxygen signaling at the consensus HRE. Sci. STKE 2005:re12 10.1126/stke.3062005re12 16234508

[B143] West X. Z. Malinin N. L. Merkulova A. A. Tischenko M. Kerr B. A. Borden E. C. (2010). Oxidative stress induces angiogenesis by activating TLR2 with novel endogenous ligands. Nature 467, 972–976 10.1038/nature09421 20927103 PMC2990914

[B144] Yachie A. Niida Y. Wada T. Igarashi N. Kaneda H. Toma T. (1999). Oxidative stress causes enhanced endothelial cell injury in human heme oxygenase-1 deficiency. J. Clin. Invest. 103, 129–135 10.1172/JCI4165 9884342 PMC407858

[B145] Yeagle P. L. (1985). Cholesterol and the cell membrane. Biochim. Biophys. Acta 822, 267–287 10.1016/0304-4157(85)90011-5 3904832

[B146] Yet S. F. Layne M. D. Liu X. Chen Y. H. Ith B. Sibinga N. E. (2003). Absence of heme oxygenase-1 exacerbates atherosclerotic lesion formation and vascular remodeling. FASEB J. 17, 1759–1761 10.1096/fj.03-0187fje 12958201

[B147] Zarjou A. Jeney V. Arosio P. Poli M. Antal-Szalmas P. Agarwal A. (2009). Ferritin prevents calcification and osteoblastic differentiation of vascular smooth muscle cells. J. Am. Soc. Nephrol. 20, 1254–1263 10.1681/ASN.2008070788 19423691 PMC2689905

[B148] Zemplenyi T. Crawford D. W. Cole M. A. (1989). Adaptation to arterial wall hypoxia demonstrated *in vivo* with oxygen microcathodes. Atherosclerosis 76, 173–179 10.1016/0021-9150(89)90101-9 2730714

[B149] Zhang Y. Cliff W. J. Schoefl G. I. Higgins G. (1993). Immunohistochemical study of intimal microvessels in coronary atherosclerosis. Am. J. Pathol. 143, 164–172 7686341 PMC1886935

